# Diagnostic and Treatment Initiation Delays Among Patients With Drug-Resistant Tuberculosis in North India: A Mixed-Methods Study

**DOI:** 10.7759/cureus.117721

**Published:** 2026-10-10

**Authors:** Sandeep Das, Rakesh Kumar, Anand Krishnan, Shashi Kant, Anant Mohan

**Affiliations:** 1 Department of Community Medicine and Family Medicine, All India Institute of Medical Sciences, Bhubaneswar, Bhubaneswar, IND; 2 Centre for Community Medicine, All India Institute of Medical Sciences, New Delhi, New Delhi, IND; 3 Department of Pulmonary, Critical Care and Sleep Medicine, All India Institute of Medical Sciences, New Delhi, New Delhi, IND

**Keywords:** delay in treatment initiation, diagnostic delay, diagnostic turnaround time, drug-resistant tuberculosis (dr-tb), mixed-methods approach, north india, turnaround time (tat)

## Abstract

Background: Delays in diagnosis and treatment initiation of drug-resistant tuberculosis (DR-TB) persist in India despite scale-up of rapid diagnostics. We estimated the magnitude and determinants of these delays and explored barriers.

Methods: This mixed-methods study was conducted (October 2021-June 2022) at three district DR-TB centres in Delhi and Faridabad, India. Diagnostic and treatment initiation turnaround times (D-TAT and TI-TAT) were calculated for 158 patients. Delays were defined per NTEP as D-TAT >15 and TI-TAT >7 days. Logistic regression assessed determinants. In-depth interviews with 16 patients/caregivers and 16 programme officials explored barriers.

Results: Median D-TAT was 24 days (IQR 7-67) and TI-TAT 5 days (IQR 3-11); 60.1% had diagnostic delay and 33.5% treatment initiation delay. Diagnostic delay was associated with line probe assay use (aOR 6.6, 95% CI 1.7-26.2) and inversely with cartridge-based nucleic acid amplification test (CBNAAT) use (aOR 0.2, 95% CI 0.1-0.9). Treatment initiation delay was associated with residence in Faridabad (aOR 3.1, 95% CI 1.4-6.4) and, imprecisely, with age ≤ 18 years (aOR 6.4, 95% CI 1.0-39.6; p = 0.045). Qualitative findings identified insufficient CBNAAT availability, intermediate reference laboratory (IRL) backlogs, poor awareness, stigma, and weak private-to-public referrals.

Conclusions: Six in 10 patients experienced diagnostic delay, and one in three experienced treatment initiation delay. Expanding rapid molecular diagnostics at peripheral levels and strengthening referral mechanisms are essential to reducing delays.

## Introduction

Drug-resistant tuberculosis (DR-TB) poses a major threat to achieving tuberculosis elimination targets globally. In 2024, an estimated 390,000 people developed multidrug-resistant or rifampicin-resistant tuberculosis (MDR/RR-TB) globally, with India accounting for 32% of the burden [1]. In 2023, the programme diagnosed 63,939 cases of MDR-TB nationally, with a treatment success rate of 65% [2]. DR-TB is associated with poorer treatment outcomes, prolonged treatment duration with second-line drugs, and substantial adverse effects, all compounded by delays in diagnosis and treatment initiation [3].

Delays in the DR-TB care pathway operate through multiple causal pathways to worsen outcomes: prolonged time to treatment initiation is associated with more than two-fold higher odds of death (with mortality falling from 30.8% to 6.9% in Rwanda as delays decreased over a decade)[4], higher sputum smear grades and resistance to drugs beyond isoniazid and rifampicin at treatment initiation [5], and approximately two-fold higher odds of acquiring resistance to second-line anti-tubercular drugs [6]. Additionally, delays extend the period of infectivity, increasing community transmission of drug-resistant strains [7-9].

Despite programmatic scale-up under India's National Tuberculosis Elimination Programme (NTEP), including expansion of cartridge-based nucleic acid amplification tests (CBNAATs) and decentralisation of DR-TB services, diagnostic delays of up to 110 days and treatment initiation delays of up to 87 days have been reported [2,3,10-21]. Factors include prolonged diagnostic pathways, bottlenecks at intermediate reference laboratories (IRLs), and poor private-to-public referral tracking [12,13]. The NTEP defines diagnostic delay as turnaround time exceeding 15 days from eligibility for drug susceptibility testing (DST) to DR-TB diagnosis, and treatment initiation delay as exceeding seven days from diagnosis to treatment start [3].

However, most Indian studies have used heterogeneous definitions, and evidence from Northern India remains limited [11-13,15,19-23]. Few studies have employed mixed-methods designs to simultaneously quantify delays and explore barriers from patient and programme perspectives. This study estimated the magnitude and determinants of diagnostic and treatment initiation delays, and explored barriers to timely care, among patients with DR-TB at district DR-TB centres in Delhi and Haryana.

## Materials and methods

Study design and setting

This was a sequential mixed-methods study comprising a cross-sectional quantitative component followed by a qualitative component. The study was conducted at three District Drug-Resistant TB Centres (DDRTBCs) in North India - one in Faridabad, Haryana, and two in the National Capital Territory of Delhi - between October 2021 and June 2022. Together, these centres served a mixed urban and semi-urban population with a combined annual DR-TB caseload of approximately 200 patients.

Study population

All patients with DR-TB newly registered under the NTEP at the selected DDRTBCs during the study period were approached for inclusion. Patients on retreatment for DR-TB, those too sick to cooperate, or those unable to comprehend the questionnaire were excluded.

Sample size

No Indian study had reported the variability of the NTEP-defined diagnostic turnaround time when the protocol was written. Hence, the estimate of Sharma et al. [24] for the interval from first healthcare facility (HCF) visit to confirmation of MDR-TB was used for calculation. The mean was 142.5 days and the standard deviation was 173.6 days. That interval contains the D-TAT interval, so its variance is no smaller, and the resulting sample size is conservative. Taking a margin of error of 28.5 days (from a relative error of 20%) and a non-response/refusal rate of 10%, the sample size was calculated to be 156 as per the formula. The final required sample size taken for the study was 156.

$n=\ \frac{{\left(Z_{\alpha /2}\times \sigma \right)}^{2}}{E^{2}}\ =\ \frac{{(1.96\times 173.6)}^{2}}{{(0.2\times 142.5)}^{2}}\approx 142\ (without\ nonresponse\ inflation) \approx 156 (after\ 10\% \ nonresponse \ inflation )$

Data collection

A structured questionnaire adapted from the World Health Organisation's "Diagnostic and Treatment Delay in Tuberculosis" tool (2006) was used [25]. Information on socio-demographic characteristics, including age, sex, education, occupation, residence, ownership of household assets, and personal habits such as tobacco and alcohol use, was obtained through face-to-face interviews with patients or their caregivers. Clinical information, including dates of symptom onset, diagnostic investigations (AFB smear microscopy, CBNAATs, line probe assay (LPA), and culture), DR-TB diagnosis, and treatment initiation, was extracted from the DR-TB treatment register and medical records maintained at the DDRTBCs; where records were unavailable, this information was obtained from patient interviews. A household wealth index was constructed using principal component analysis based on ownership of household assets (television, refrigerator, washing machine, motorcycle, car), type of flooring, toilet facility, and source of drinking water; the first principal component score was used to classify households into wealth terciles (poorest, middle, richest).

Definitions of turnaround times and delays

Time intervals are defined in Figure 1. Diagnostic turnaround time (D-TAT) was defined as the interval from eligibility for DST to DR-TB diagnosis; for smear-positive patients, eligibility was the date of smear examination, and for smear-negative patients, the date of drug-sensitive TB treatment initiation. Each diagnostic test was coded as performed if its result was available on or before the date of DR-TB diagnosis, so that the exposure window does not extend beyond the end of D-TAT. Treatment initiation turnaround time (TI-TAT) was defined as the interval from DR-TB diagnosis to treatment initiation. Diagnostic delay was defined as D-TAT >15 days and treatment initiation delay as TI-TAT >7 days, per NTEP PMDT guidelines [3]. Care-seeking interval was defined as the time from symptom onset to the first health facility visit, and pre-diagnostic interval as the time from the first health facility visit to DR-TB diagnosis.

**Figure 1:**
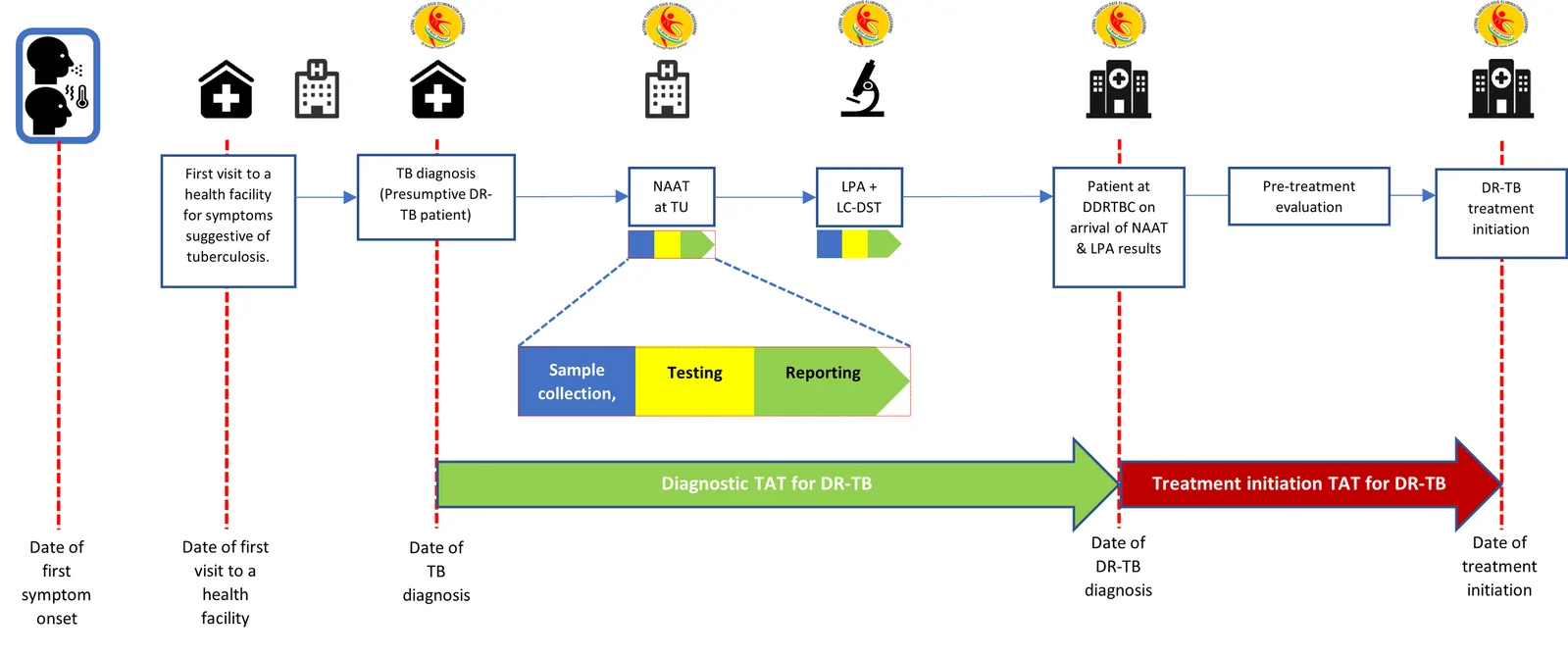
Conceptual framework of turnaround times corresponding to the integrated diagnostic and treatment algorithm for drug-resistant tuberculosis by NTEP. Created in Microsoft PowerPoint (Microsoft Corporation, Redmond, USA). NTEP: National Tuberculosis Elimination Programme

Statistical analysis

Analysis was performed using Stata 17.0 (StataCorp, College Station, TX). Turnaround times were reported as medians with interquartile ranges (IQRs). Bivariate logistic regression was performed to identify factors associated with delays; variables with p<0.2 were included in multivariable models and statistical significance was set at 5%. This threshold was chosen so that potentially relevant covariates were not discarded. Because both models estimated a large number of parameters relative to the number of outcome events, two pre-specified sensitivity analyses were performed: parsimonious models restricted to covariates with an a priori rationale, and Firth penalised-likelihood re-estimation of the full models. Both are reported in the supplementary appendix. Each diagnostic test whose result was available on or before the date of DR-TB diagnosis (AFB microscopy, CBNAAT, LPA, culture) was entered as an independent binary variable (yes/no) in the multivariable model. Given the number of covariates assessed relative to sample size, the risk of type I error from multiple comparisons was considered when interpreting individual associations.

Qualitative component

Patients with DR-TB or their caregivers (n=16) not included in the quantitative component, and health system stakeholders (n=16) including the State TB Officer, District TB Officers, Medical Officers-TB Control, Senior Treatment Supervisors, Senior TB Laboratory Supervisors, DOTS-Plus treatment supervisors (DOTS = Directly Observed Treatment Short-course), and a microbiologist at an Intermediate Reference Laboratory were interviewed using in-depth interview guides. Purposive sampling was used. Interviews were audio-recorded, transcribed verbatim in English, and analysed using deductive and inductive coding in ATLAS.ti (Version 7.5.18). Themes were identified and presented using a conceptual framework adapted from the patient-centred access model by Levesque et al. and the mixed-methods framework for DR-TB delays by Omenka et al. [26,27].

Ethical considerations

Ethical approval was obtained from the AIIMS Institutional Ethics Committee (IECPG-748/23.12.2021). Written informed consent was obtained from all participants prior to enrolment.

## Results

Patient characteristics

Of 192 patients on treatment for DR-TB, 158 were enrolled (17 not contactable, five died, six lost to follow-up, six refused). The mean age was 28.9 (SD 13.7) years; 55.7% were female. Among enrolled patients, 84.2% had pulmonary DR-TB and 75.3% had MDR/RR-TB. Only 22.2% had an LPA result and 1.3% a culture result available by the date of DR-TB diagnosis. Table 1 describes the patient characteristics in detail.

**Table 1:** Characteristics of patients with drug-resistant tuberculosis enrolled from Delhi and Faridabad, India. DR-TB: Drug-resistant tuberculosis; MDR/RR-TB: multidrug-resistant or rifampicin-resistant tuberculosis; CBNAAT: cartridge-based nucleic acid amplification test

Variable	Category (N = 158)	Frequency (%)
Age group	≤18 years	42 (26.6)
	19-30 years	59 (37.3)
	31-50 years	43 (27.2)
	>50 years	14 (8.9)
Sex	Male	70 (44.3)
Residence	Rural	23 (14.6)
	Urban	135 (85.4)
Region	Delhi	79 (50)
	Faridabad	79 (50)
Religion	Hindu	137 (86.7)
	Others	21 (13.3)
Years of education	≤5 years	44 (27.9)
	6-12 years	85 (53.8)
	>12 years	29 (18.3)
Occupation	Formal employees and sales workers	24 (15.1)
	Manual Workers & Elementary Occupations	38 (24.1)
	Unemployed (including students and housewives)	96 (60.8)
Monthly family income (pre-TB) (Median (IQR), in USD)		204.4 (144.3 – 360.7)
Ever use of smoking tobacco		29 (18.3)
Ever use of smokeless tobacco		22 (13.9)
Ever use of alcohol		29 (18.3)
Wealth Index (in terciles)	I (Poorest)	53 (33.5)
	II	53 (33.5)
	III (Richest)	52 (33.0)
Site of TB	Pulmonary	133 (84.2)
	Extra-pulmonary	25 (15.8)
Type of DR-TB case	H mono/poly DR-TB	19 (12.0)
	MDR/RR-TB	119 (75.3)
	Pre XDR-TB	14 (8.9)
	XDR-TB	6 (3.8)
Treatment regimen	H mono/poly regimen	17 (10.8)
	Shorter Oral Bdq-containing MDR/RR-TB regimen	46 (29.1)
	Longer oral M/XDR-TB regimen	92 (58.2)
	BPaL regimen	3 (1.9)
History of TB	New (No past episode of TB)	107 (67.7)
	Retreatment (≥1 past episodes of TB)	51 (32.3)
Number of episodes of TB in past	0	107 (67.7)
	1	45 (28.5)
	≥2	6 (3.8)
Diagnostic tests with results available by the date of DR-TB diagnosis		
AFB microscopy		63 (39.9)
CBNAAT		130 (82.3)
Line probe assay		35 (22.2)
Culture		2 (1.3)

Table 2 describes the distribution and time durations of symptoms of DR-TB patients. The most common symptoms were fever (86.7%), fatigue (82.9%), weight loss (77.2%), and cough (75.3%). Haemoptysis had the shortest median duration before diagnosis (28 days, IQR 7-56) and enlarged lymph nodes the longest (81 days, IQR 46-218).

**Table 2:** Distribution and time durations of symptoms of patients with drug-resistant tuberculosis enrolled from Delhi and Faridabad, India. DR-TB: Drug-resistant tuberculosis

Symptom	Patients presenting (n(n/N%), N=158)	Duration before DR-TB diagnosis (days)	
		Mean (SD)	Median (IQR)
Fever	137 (86.7)	128.3 (169.8)	77 (42 – 147)
Fatigue	131 (82.9)	113.9 (121.3)	70 (42 – 135)
Weight loss	122 (77.2)	129.2 (173.9)	70 (40 – 148)
Cough	119 (75.3)	124.6 (170.6)	77 (43 – 125)
Night sweats	115 (72.8)	124.5 (173.0)	76 (42 – 135)
Loss of appetite	115 (72.8)	114.0 (112.6)	77 (43 – 135)
Chest pain	78 (49.4)	119.4 (187.2)	72 (39 – 125)
Enlarged lymph nodes	24 (15.2)	134.0 (193.0)	81 (46 – 218)
Haemoptysis	23 (14.6)	66.1 (107.6)	28 (7 – 56)

Figure 2 illustrates the distribution of diagnostic and treatment initiation delays among 158 patients with DR-TB, with their median turnaround times. The median care-seeking interval was 20 days (IQR 9-39), median pre-diagnostic interval was 10 days (IQR 2-32), median D-TAT was 24 days (IQR 7-67), median TI-TAT was five days (IQR 3-11), and median total duration from symptom onset to treatment initiation was 89 days (IQR 51-171). Forty-two patients (26.6%) experienced neither diagnostic nor treatment initiation delay; 63 (39.9%) had only diagnostic delay (median D-TAT 63 days, median TI-TAT three days); 21 (13.3%) had only treatment initiation delay (median D-TAT 5 days, median TI-TAT 14 days); and 32 (20.3%) had both (median D-TAT 48 days, median TI-TAT 17 days). The basis of the DR-TB diagnosis differed between patients. In 110 patients (69.6%), the diagnosis rested on CBNAAT alone, with no LPA result available on the date of diagnosis. In 35 patients (22.2%), the diagnosis awaited the line probe assay report. In 13 patients (8.2%), neither result was available at the study centre on the date of diagnosis; 11 of these first presented at a private facility. Median laboratory turnaround from sample collection to result was 2 days (IQR 1-4) for CBNAAT and 17 days (IQR 8-30) for the LPA. Median D-TAT was 14 days (IQR 5-45) where diagnosis rested on CBNAAT alone and 63 days (IQR 22-101) where it awaited the line probe assay. The same breakdown, with the corresponding turnaround times, is given in Appendix 1.

**Figure 2:**
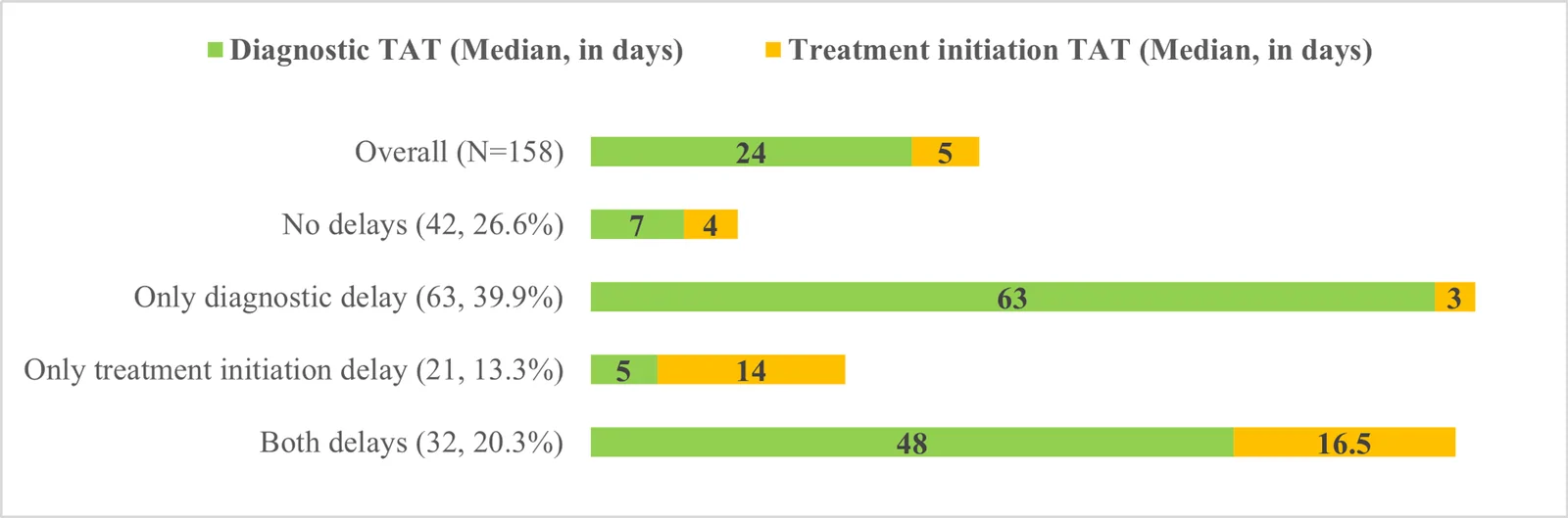
Distribution of diagnostic and treatment initiation delays among 158 patients with drug-resistant tuberculosis, with their median turnaround times. Grouped bars show median diagnostic turnaround time (D-TAT, in days; green) and median treatment initiation turnaround time (TI-TAT, in days; yellow) for each subgroup. Medians are presented side-by-side rather than stacked, as the sum of two medians does not equal the median of their sum. Created in Microsoft PowerPoint (Microsoft Corporation, Redmond, USA).

In bivariate analysis for determinants of diagnostic delay, potentially significant factors (p<0.2) included age, region, site of TB, history of smoking and smokeless tobacco use, AFB microscopy, CBNAAT, LPA, and symptoms including cough, haemoptysis, chest pain, fever, weight loss, and enlarged lymph nodes. On multivariable analysis, patients with history of smokeless tobacco consumption had four-fold lower odds of diagnostic delay compared to those who didn't consume smokeless tobacco (aOR 0.25, 95% CI: 0.1-0.8). A CBNAAT result available on or before the date of DR-TB diagnosis was associated with lower odds of diagnostic delay (aOR 0.23, 95% CI 0.06-0.86). A LPA result available on or before that date was associated with nearly seven-fold higher odds (aOR 6.64, 95% CI 1.68-26.22) (Table 3).

**Table 3:** Factors associated with diagnostic delay among patients with drug-resistant tuberculosis in three selected DDRTBCs in Delhi and Faridabad. ^*^p-value <0.2, ^#^p-value <0.05. Culture was omitted from the multivariable model owing to perfect prediction (both patients with a culture result available by the date of DR-TB diagnosis experienced diagnostic delay). DR-TB: Drug-resistant tuberculosis; DDRTBCs: District Drug-Resistant TB Centres; CBNAAT: cartridge-based nucleic acid amplification test; LPA: line probe assay

Variable		Total (N)	Diagnostic delay (n(%N))	Bivariate analysis	Multivariable analysis
				cOR (95% CI)	aOR (95% CI)
Age group	≤18 years	42	27 (64.3)	3.2 (0.9 – 11.5)*	2.7 (0.5 – 15.3)
	19-30 years	59	38 (64.4)	3.2 (1.0 – 11.0)*	2.4 (0.5 – 12.0)
	31-50 years	43	25 (58.1)	2.5 (0.7 – 8.7)*	3.2 (0.6 – 15.9)
	>50 years	14	5 (35.7)	Ref.	Ref.
Sex	Female	88	53 (60.23)	Ref.	Not included
	Male	70	42 (60.0)	1.0 (0.5 – 1.9)	
Residence	Rural	23	16 (69.6)	Ref.	Not included
	Urban	135	79 (58.5)	0.6 (0.2 – 1.6)	
Region	Delhi	79	53 (67.1)	Ref.	Ref.
	Faridabad	79	42 (53.1)	0.5 (0.3 – 1.1)*	1.4 (0.6 – 3.4)
Religion	Hindu	137	84 (61.3)	Ref.	Not included
	Other	21	11 (52.4)	0.7 (0.3 – 1.8)	
Wealth Index	I (Poorest)	53	33 (62.3)	Ref.	Not included
	II	53	32 (60.4)	0.9 (0.4 – 2.0)	
	III (Richest)	52	30 (57.7)	0.8 (0.4 – 1.8)	
Years of education	≤5 years	44	23 (52.3)	Ref.	Not included
	6-12 years	85	53 (62.4)	1.5 (0.7 – 3.2)	
	>12 years	29	19 (65.5)	1.7 (0.7 – 4.6)	
Years of education (for every 1-year increase)				1.03 (0.9 – 1.10)	Not included
Occupation	Unemployed (including students and housewives)	96	58 (60.4)	Ref.	Not included
	Formal employees and sales workers	24	13 (54.2)	0.8 (0.3 – 1.9)	
	Manual Workers & Elementary Occupations	38	24 (63.2)	1.1 (0.5 – 2.4)	
Site of TB	Pulmonary	133	75 (56.4)	Ref.	Ref.
	Extra-pulmonary	25	20 (80.0)	3.1 (1.1 - 8.7)*	2.3 (0.5 – 11.0)
Ever did smoking	Yes	29	14 (48.3)	0.6 (0.2–1.2)*	0.9 (0.3–2.8)
	No	129	81 (62.8)	Ref.	Ref.
Ever used smokeless tobacco	Yes	22	8 (36.4)	0.3 (0.1–0.8)*	0.25 (0.1–0.8)^#^
	No	136	87 (64.0)	Ref.	Ref.
History of alcohol	Yes	29	15 (51.7)	0.7 (0.3 – 1.5)	Not included
	No	129	80 (62.0)	Ref.	
Number of episodes of TB in past	0	107	62 (57.9)	Ref.	Not included
	1	45	31 (68.9)	1.6 (0.8 – 3.4)	
	≥2	6	2 (33.3)	0.4 (0.1 – 2.1)	
First facility visited	Govt.	80	46 (57.5)	Ref.	Not included
	Pvt.	78	49 (62.8)	1.3 (0.7 – 2.4)	
Diagnostic tests with results available by the date of DR-TB diagnosis (Ref. – “No”)	AFB microscopy	63	44 (69.8)	2.0 (1.0 – 3.9)*	2.3 (0.9 – 5.4)
	CBNAAT	130	71 (54.6)	0.2 (0.1 – 0.6)*	0.2 (0.1 – 0.9)^#^
	LPA	35	32 (91.4)	10.1 (3.0 – 34.9)*	6.6 (1.7 – 26.2)^#^
	Culture	2	2 (100)	1 (omitted)	Not included
Symptoms (if present in patient)	Cough	119	67 (56.3)	0.5 (0.2 – 1.1)*	0.6 (0.2 – 2.1)
	Haemoptysis	23	11 (47.9)	0.6 (0.2 – 1.4)*	0.6 (0.2 – 2.1)
	Night sweats	115	66 (57.4)	0.6 (0.3 – 1.4)	Not included
	Chest pain	78	42 (53.9)	0.6 (0.3 – 1.1)*	0.5 (0.2 – 1.3)
	Fever	137	79 (57.7)	0.4 (0.2 – 1.2)*	0.3 (0.1 – 1.3)
	Weight loss	122	78 (63.9)	2.0 (0.9 – 4.2)*	3.1 (1.0 – 9.8)
	Loss of appetite	115	71 (61.7)	1.3 (0.6 – 2.6)	Not included
	Fatigue	131	79 (60.3)	1.0 (0.5 – 2.4)	Not included
	Enlarged lymph nodes	24	19 (79.2)	2.9 (1.0 – 8.2)*	1.1 (0.2 – 5.9)

In bivariate analysis for determinants of treatment initiation delay, potentially significant factors included age, region, years of education, occupation, and site of TB. On multivariable analysis, patients aged 18 years or younger had six-fold higher odds of treatment initiation delay compared to those above 50 years (aOR 6.4, 95% CI: 1.0-39.6), and patients residing in Faridabad had three-fold higher odds compared to those in Delhi (aOR 3.1, 95% CI: 1.5-6.7) (Table 4).

**Table 4:** Factors associated with treatment initiation delay among patients with drug-resistant tuberculosis in three selected DDRTBCs in Delhi and Faridabad ^*^p-value <0.2, ^#^p-value <0.05 DDRTBCs: District Drug-Resistant TB Centres; XDR-TB: extensively drug-resistant tuberculosis; MDR/RR-TB: multidrug-resistant or rifampicin-resistant tuberculosis

Variable		Total (N)	Treatment initiation delay (n (%N))	Bivariate analysis	Multivariable analysis
				cOR (95% CI)	aOR (95% CI)
Age	≤ 18 years	42	19 (45.2)	5.0 (1.0 – 24.9)*	6.4 (1.0 – 39.6)^#^
	19-30 years	59	18 (30.5)	2.6 (0.5 – 13.0)	3.95 (0.69 – 22.7)
	31-50 years	43	14 (32.6)	2.9 (0.6 – 14.7)*	4.4 (0.8 – 25.5)
	>50 years	14	2 (14.3)	Ref.	Ref.
Sex	Female	88	31 (35.2)	Ref.	Not included
	Male	70	22 (31.4)	0.8 (0.4 – 1.6)	
Region	Delhi	79	20 (25.3)	Ref.	Ref.
	Faridabad	79	33 (41.8)	2.1 (1.1 – 4.2)*	3.1 (1.5 – 6.7)^#^
Residence	Rural	23	9 (39.1)	Ref.	Not included
	Urban	135	44 (32.6)	0.8 (0.3 – 1.9)	
Religion	Hindu	137	45 (32.9)	Ref.	Not included
	Others	21	8 (38.1)	1.3 (0.5 – 3.3)	
Years of Education	≤5 years	44	15 (34.1)	Ref.	Ref.
	6-12 years	85	31 (36.5)	1.1 (0.5 – 2.4)	0.9 (0.4 – 2.1)
	>12 years	29	7 (24.1)	0.3 (0.1 – 1.7)*	0.4 (0.1 – 1.4)
Years of education (for every 1-year increase)				1.0 (0.9 – 1.1)	Not included
Wealth Index	I (Poorest)	53	15 (28.3)	Ref.	Not included
	II	54	21 (38.9)	1.6 (0.7 – 3.6)	
	III (Richest)	51	17 (33.3)	1.3 (0.6 – 2.9)	
Site of TB	Pulmonary	133	41 (30.8)	Ref.	Ref.
	Extra-pulmonary	25	12 (48.0)	2.1 (0.9 – 4.9)	2.5 (1.0 – 6.5)
Type of DR-TB case	H mono/poly DR-TB	19	6 (31.6)	Ref.	Not included
	MDR/RR-TB	119	37 (31.1)	1.0 (0.3 – 2.8)	
	Pre XDR-TB	14	7 (50.0)	2.2 (0.5 – 9.0)	
	XDR-TB	6	3 (50.0)	2.2 (0.3 – 14.1)	
Regimen	BPaL	3	2 (66.7)	Ref.	Not included
	H mono/poly	17	5 (29.4)	0.2 (0.0 – 2.9)	
	Longer oral M/XDR-TB	92	28 (30.4)	0.2 (0.0 – 2.5)	
	Shorter oral Bdq MDR/RR-TB	46	18 (39.1)	0.3 (0.0 - 3.8)	
Occupation	Unemployed (including students and housewives)	96	37 (38.5)	Ref.	Ref.
	Formal employees and sales workers	24	6 (25.0)	0.5 (0.2 – 1.5)	0.7 (0.2 – 2.2)
	Manual Workers & Elementary Occupations	38	10 (26.3)	0.6 (0.3 – 1.3)	0.5 (0.2 – 1.5)

Twenty-six distinct care-seeking pathways were identified; 50.6% initially sought care at a public facility, but only 5.1% reached the DDRTBC directly (Table 5).

**Table 5:** Care seeking pathway before initiation of treatment among patients with drug-resistant tuberculosis in the three selected DDRTBCs in Delhi and Faridabad G: Government health facility; P: Private health facility; DDRTBCs: District Drug-Resistant TB Centres

Pathways till start of DR-TB treatment	Number of patients (%) (N = 158)
G --> DDRTBC	35 (22.2%)
P --> G --> DDRTBC	30 (19.0%)
G --> DDRTBC --> G	18 (11.4%)
P --> DDRTBC	18 (11.4%)
P --> DDRTBC --> G	11 (7.0%)
G --> DDRTBC --> G --> DDRTBC	10 (6.3%)
P --> DDRTBC --> G --> DDRTBC	7 (4.4%)
P --> G --> DDRTBC --> G	4 (2.5%)
G --> P --> DDRTBC	3 (1.9%)
DDRTBC --> G --> DDRTBC	2 (1.3%)
DDRTBC --> P --> DDRTBC	2 (1.3%)
G --> DDRTBC --> G --> DDRTBC --> G	2 (1.3%)
P --> Outside NCR --> G --> DDRTBC --> G	2 (1.3%)
P --> G --> DDRTBC --> G --> DDRTBC	2 (1.3%)
Outside NCR --> G --> DDRTBC --> G --> DDRTBC	1 (0.6%)
Outside NCR --> P --> G --> DDRTBC --> G --> DDRTBC	1 (0.6%)
DDRTBC	1 (0.6%)
DDRTBC --> G	1 (0.6%)
DDRTBC --> G --> DDRTBC --> G	1 (0.6%)
DDRTBC --> G --> P --> G --> DDRTBC	1 (0.6%)
G --> DDRTBC --> Outside NCR --> DDRTBC --> G --> DDRTBC --> G	1 (0.6%)
G --> P --> DDRTBC --> G	1 (0.6%)
G --> P --> G --> DDRTBC	1 (0.6%)
G --> P --> G --> DDRTBC --> G	1 (0.6%)
P --> DDRTBC --> G --> DDRTBC --> G --> DDRTBC	1 (0.6%)
P --> G --> DDRTBC --> G --> DDRTBC --> G	1 (0.6%)

Qualitative study

Interviews were conducted by the first author, a male physician trained in qualitative research methods, who had no prior clinical relationship with the participants. Separate pre-piloted topic guides were used for patients or caregivers and for health-system stakeholders. Patients and caregivers were interviewed in Hindi, at a location of their choosing. Stakeholders were interviewed in Hindi or English, according to preference. All interviews were audio-recorded with written consent and supported by verbatim field notes. Recordings were transcribed and translated into English by the interviewer. Thirty-two transcripts were independently back-checked against the original recordings by an independent reviewer, to confirm that meaning had been preserved. Transcripts were not returned to participants for comment.

Coding was both deductive and inductive. The deductive framework came from the patient-centred access model by Levesque et al. [26] and the mixed-methods framework for DR-TB delays by Omenka et al. [27]. Inductive codes were added as they arose. Coding was done in ATLAS.ti version 7.5.18 by two coders working independently. Disagreements were resolved by adjudication by a third reviewer. Themes were checked back against the original transcripts. Purposive sampling continued until no new codes were generated; the final interviews in each group produced no new codes, which we took as thematic saturation. Sixteen patients or caregivers (eight at each of two DDRTBCs) and 16 health-system stakeholders, with seven to 10 years of programme experience, were interviewed. Findings are presented in Figure 3 as patient-level and health-system factors, organised along the domains of the Levesque et al. access framework [26]. Illustrative quotes are presented in Table 6.

**Figure 3:**
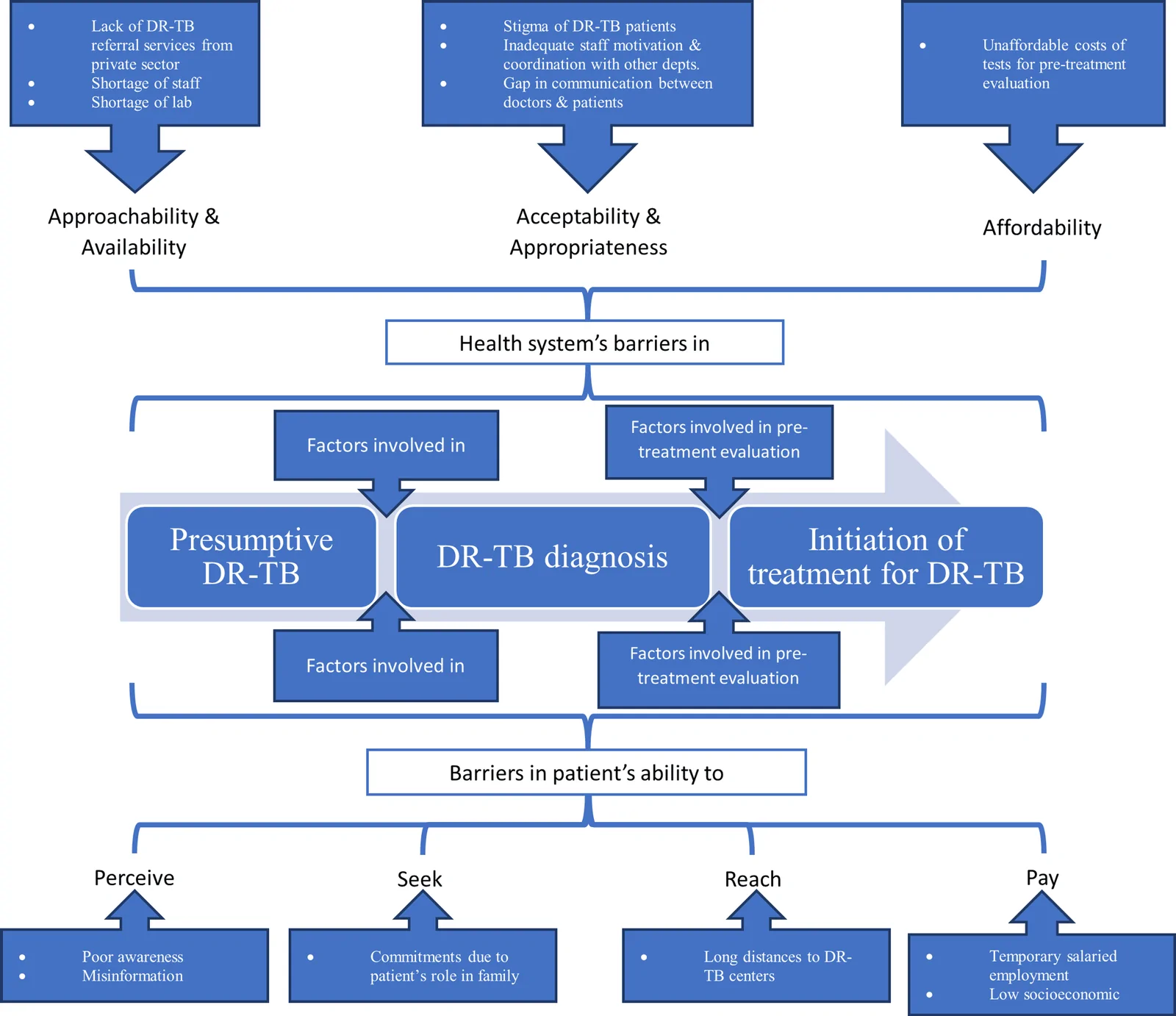
Conceptual framework of reasons causing delays in diagnosis and treatment initiation in drug-resistant tuberculosis. Created in Microsoft PowerPoint (Microsoft Corporation, Redmond, USA).

**Table 6:** Illustrative quotes from in-depth interviews with patients/caregivers and health system stakeholders, organised by domains of the patient-centred access framework by Levesque et al. DR-TB: Drug-resistant tuberculosis; CBNAAT: cartridge-based nucleic acid amplification test

Access framework domain	Subtheme	Respondent	Illustrative quote
Patient-level factors	Ability to perceive need for care	32-year-old male patient, Faridabad	“I didn’t know anything about TB. I had fever every evening, no appetite, joint pains all over. I thought I had typhoid, but at the hospital I found out it was TB.”
	Ability to seek care	30-year-old male patient, Delhi	“My health was bad, but I couldn’t take leave — my boss deducts a full day’s salary even for a short absence.”
	Ability to reach care	32-year-old patient, Delhi	“We had to go to AIIMS, which is far from our house, to give samples. There’s a lot of expense in going and coming, and one must take a whole day’s leave.”
	Ability to pay	28-year-old patient, Faridabad	“Due to frequent visits to doctors, I didn’t get my salary on time. Managing household expenses became very difficult.”
Health-system factors	Approachability and availability	DOTS Coordinator	“Not all tuberculosis units have CBNAAT/TrueNAT machines. Samples sent to intermediate reference laboratories take one to two months for results, and during COVID there were reagent shortages as well.”
	Acceptability and appropriateness	Senior Treatment Supervisor	“Patients come to us carrying CBNAAT reports without understanding their diagnosis. Doctors at private facilities send them without clearly telling them they have DR-TB.”
	Affordability	District Tuberculosis Officer	“Out-of-pocket expenses for these patients are terrible… sometimes patients are unable to afford even the subsidised rates of laboratory tests.”

Patient-Level factors

Poor awareness and misinformation about tuberculosis hindered recognition of the need for care despite characteristic symptoms. Even after recognising symptoms, patients trivialised the illness; competing work and family commitments and fear of wage loss took precedence over care-seeking. Stigma, particularly concerns about marriage prospects of young female patients, further reduced care-seeking. Patients described limited awareness of nearby public health facilities and long distances to diagnostic centres, with multiple referrals between private and public facilities prolonging the pathway. Substantial out-of-pocket expenditure on travel, pre-treatment investigations, and wage loss compounded these barriers.

Health-System factors

Not all tuberculosis units had CBNAAT/TrueNAT machines, and samples sent to IRLs experienced reporting delays of one to two months, exacerbated by reagent shortages during the COVID-19 pandemic. Unfilled staff vacancies increased workload. Physicians at private facilities often did not clearly communicate the DR-TB diagnosis, leading to confusion and delayed referral. Private-to-public referral rates were estimated at only 10-20%. Although TB diagnosis and treatment are free under NTEP, patients still incurred costs for pre-treatment evaluation tests not always available at government facilities.

## Discussion

Significant delays in diagnosis and treatment initiation were found among patients with drug-resistant tuberculosis enrolled in the NTEP. We found that 60% of patients experienced diagnostic delay and one-third experienced treatment initiation delay, with a median of 89 days from symptom onset to treatment initiation. Qualitative findings revealed convergent barriers at both patient and health-system levels, with the triangulation of quantitative determinants and qualitative explanations strengthening the inference that demand-side and supply-side factors operate synergistically to produce these delays.

The median D-TAT of 24 days (IQR 7-67) in our study is lower than that reported by Nair et al. in a 2012 Indian cohort [28], likely reflecting improvements in the diagnostic cascade following the introduction of molecular diagnostic tests and the benchmarks set by the 2021 PMDT Guidelines [3]. However, it remains higher than the 15-day cut-off stipulated by these guidelines and is comparable to findings from studies in Ethiopia, Bangladesh, and China [14,17,18]. Diagnostic delay was inversely associated with CBNAAT use (aOR 0.2) and directly with LPA use (aOR 6.6), consistent with the rapid turnaround of CBNAAT versus the sample transport and IRL backlogs required for LPA, as corroborated by our qualitative findings and consistent with Ethiopian data [14]. These associations reflect the structural positioning of these tests within the NTEP diagnostic algorithm rather than patient-level risk factors: CBNAAT provides point-of-care molecular diagnosis at the tuberculosis unit, whereas LPA requires sample transport to an intermediate reference laboratory and batch processing. Because rifampicin resistance on CBNAAT is itself sufficient to register RR-TB, whereas diagnosis was held for the line probe assay where isoniazid resistance mattered, or the CBNAAT result was discordant, the line probe assay variable identifies patients whose diagnosis date was set by that report; its laboratory turnaround therefore falls partly inside the diagnostic turnaround time by construction, and the association should not be read as an effect of the test. The association of non-use of smokeless tobacco with diagnostic delay (aOR 4.0) is counterintuitive and warrants cautious interpretation. A plausible explanation is that disclosed tobacco use heightens clinical suspicion of tuberculosis, prompting clinicians to expedite testing; however, this cannot be tested with the available data. Given the number of predictors assessed relative to sample size, this finding does not survive a Bonferroni-adjusted threshold and is therefore presented as a potential chance finding requiring confirmation in larger studies.

The median care-seeking interval of 20 days and pre-diagnostic interval of 10 days were consistent with other Indian studies [20,29]. The pre-diagnostic interval has not been reported separately by previous DR-TB studies, representing a measurable programmatic indicator. Patients with non-alarming symptoms such as fever, fatigue, and weight loss had these for prolonged periods (median 70-77 days) before diagnosis, suggesting normalisation by patients or insufficient clinical suspicion, whereas haemoptysis prompted rapid care-seeking (median 28 days), consistent with findings from other high-burden settings [4,10,13,17-21,26].

The median TI-TAT of 5 days (IQR 3-11) is comparable to that in other Indian studies [13,15,20,23,28]. Treatment initiation delay was associated with residence in Faridabad (aOR 3.1, 95% CI 1.4-6.4, p = 0.003) and with younger age (≤ 18 years; aOR 6.4, 95% CI 1.0-39.6, p = 0.045). The paediatric estimate is highly imprecise. The > 50-year reference group contributed only two delayed events, and on Firth penalised re-estimation the association did not reach conventional significance (aOR 5.2, 95% CI 0.96-28.04, p = 0.055). It should therefore be read as a signal requiring confirmation in a dedicated paediatric DR-TB cohort, not as an established association. 

The qualitative component provided explanatory depth to these quantitative findings, revealing how patient-level barriers, poor awareness, competing livelihood priorities, stigma, and out-of-pocket expenditure, and health-system barriers, insufficient CBNAAT/TrueNAT availability at peripheral units, IRL backlogs exacerbated by COVID-19 reagent shortages, unfilled staff vacancies, poor diagnostic communication by private physicians, and low private-to-public referral rates, intersect and compound one another to produce the observed delays. These findings are consistent with studies from India and other high-burden settings that have identified similar barriers in the DR-TB care pathway [4,10,12,13,17-21,26].

These findings point to several measures that may warrant evaluation. These are wider availability of CBNAAT and TrueNAT at peripheral health units and decentralised LPA capacity, which the observed associations and the reported laboratory bottlenecks suggest may help reduce diagnostic delays; stronger private-to-public referral linkages through Patient Provider Support Agencies; flexible clinic timings; community engagement initiatives such as the TB Champions Campaign [30]; and streamlined pre-treatment evaluation protocols for paediatric DR-TB. Whether any of these measures do reduce delay would need to be established prospectively.

The mixed-methods design enabled triangulation of quantitative determinants with patient and health-system perspectives, and the use of NTEP-defined cut-offs enhances comparability with programmatic targets. However, there are some limitations. The sample size was calculated for estimating mean D-TAT, and both multivariable models fell below 10 outcome events per estimated parameter - 3.9 for diagnostic delay (95 events, 63 non-events, 16 parameters) and 5.9 for treatment initiation delay (53 events, nine parameters). The adjusted estimates are therefore unstable and several confidence intervals are wide, and the multivariable models should be read as exploratory. Recruitment from DDRTBCs means that presumptive patients who sought care exclusively from private facilities were not captured. Recall bias may have affected self-reported dates of symptom onset, though extraction of the majority of clinical dates from records minimises its impact. Generalisability may be limited to settings with similar health system configurations. Given the number of predictors assessed in the multivariable models relative to the sample size, type I error from multiple comparisons cannot be excluded for secondary findings; the counterintuitive protective association of smokeless tobacco use with diagnostic delay, which did not reach a Bonferroni-adjusted significance threshold, should therefore be interpreted with particular caution. Parsimonious and Firth penalised-likelihood re-estimations of both models are reported in the Supplementary Appendix; associations not reproduced across these specifications, including the paediatric treatment-initiation association, should be regarded as provisional. Although nearly half of our sample (49.4%) initially sought care in the private sector, presumptive DR-TB patients managed entirely in the private sector - or those exiting the care cascade before reaching a DDRTBC - were not observed; among enrolled patients, those who began care in the private sector had numerically longer diagnostic turnaround times (median 26.5 versus 21.5 days), suggesting that population-level diagnostic delays are likely understated by our estimates. Our D-TAT definition uses the date of drug-sensitive TB treatment initiation as the eligibility start for smear-negative patients, in line with NTEP guidelines; this captures programmatic performance but may understate the total diagnostic delay experienced by patients from the perspective of symptom onset.

## Conclusions

In this study, diagnostic and treatment initiation delays remain highly prevalent among patients with DR-TB. Wider availability of rapid molecular diagnostics at peripheral health units, decentralised LPA capacity and stronger private-to-public referral mechanisms may help reduce these delays and merit prospective evaluation.

## Appendices

Supplementary appendix

Sensitivity analyses prepared in response to peer review. All models were estimated on the full analysis dataset (N = 158) in Stata 17.0. The do-files that reproduce every table are available from the corresponding author.

Appendix 1 

Parsimonious Multivariable Models

These models are post-hoc. Their covariates were chosen on clinical grounds rather than by the data, which raises the ratio of outcome events to estimated parameters.

**Table 7:** Diagnostic delay parsimonious model N = 158; events = 95; non-events = 63; parameters = 7; events per variable = 9.0.

Variable	aOR	95% CI	p-value
Age ≤18 years (ref: >50 years)	2.8	0.65–11.96	0.166
Age 19–30 years	2.75	0.69–10.98	0.151
Age 31–50 years	2.25	0.53–9.48	0.27
Residence in Faridabad (ref: Delhi)	0.77	0.37–1.62	0.498
Extrapulmonary site (ref: pulmonary)	3.52	1.17–10.59	0.025
CBNAAT performed	0.25	0.07–0.83	0.024
Line probe assay performed	7.34	2.03–26.50	0.002

Appendix 2

Diagnostic-test exposures here are the main-analysis ones: test result available on or before the date of DR-TB diagnosis. This table therefore isolates the effect of using fewer covariates. Under the collection-date definition the same estimates are CBNAAT 0.35 (95% CI 0.11-1.11, p = 0.075) and line probe assay 4.56 (95% CI 1.80-11.53, p = 0.001).

**Table 8:** Treatment initiation delay parsimonious model N = 158; events = 53; non-events = 105; parameters = 5; events per variable = 10.6.

Variable	aOR	95% CI	p-value
Age ≤18 years (ref: >50 years)	6.95	1.30–37.21	0.024
Age 19–30 years	2.99	0.59–15.20	0.188
Age 31–50 years	3.62	0.68–19.37	0.133
Residence in Faridabad (ref: Delhi)	2.9	1.39–6.03	0.004
Extrapulmonary site (ref: pulmonary)	2.12	0.85–5.27	0.106

Appendix 3

Basis of the DR-TB Diagnosis and the Diagnostic-Test Exposure Definition

Every diagnostic-test variable in the main analysis is coded as performed if its result was available on or before the date of DR-TB diagnosis. This rule matches all four variables exactly for all 158 patients. The exposure window therefore does not extend beyond the end of the diagnostic-delay outcome.

**Table 9:** Basis of the DR-TB diagnosis DR-TB: Drug-resistant tuberculosis; CBNAAT: cartridge-based nucleic acid amplification test; D-TAT: diagnostic turnaround time

Basis of diagnosis	n (%)	Median D-TAT, days (IQR)	Diagnostic delay, n (%)
CBNAAT alone; no line probe assay result at diagnosis	110 (69.6)	14 (5–45)	52 (47.3)
Diagnosis awaited the line probe assay report	35 (22.2)	63 (22–101)	32 (91.4)
Neither result available at the study centre at diagnosis	13 (8.2)	41 (18–170)	11 (84.6)

Under the NTEP algorithm, rifampicin resistance on CBNAAT is sufficient to diagnose and register RR-TB. Diagnosis was held for the LPA where isoniazid resistance mattered or the CBNAAT result was discordant or indeterminate. Where both results were available at diagnosis (n = 20), the line probe assay was the later of the two in every case. Eleven of the 13 patients with neither result at diagnosis first presented at a private facility and arrived with a diagnosis made elsewhere. Median laboratory turnaround from sample collection to result was 2 days (IQR 1-4) for CBNAAT and 17 days (IQR 8-30) for the line probe assay. For the group whose diagnosis awaited the line probe assay, that turnaround contributes a median of 17 of the 63 days of D-TAT, about 27%, with the remaining 46 days attributable to sample transport, referral and registration.

**Table 10:** Main analysis — test result available on or before the date of DR-TB diagnosis N = 158; events = 95; non-events = 63; parameters = 16; events per variable = 3.9. DR-TB: Drug-resistant tuberculosis; CBNAAT: cartridge-based nucleic acid amplification test

Variable	aOR	95% CI	p-value
Age ≤18 years (ref: >50 years)	2.7	0.48–15.30	0.261
Age 19–30 years	2.42	0.49–11.95	0.279
Age 31–50 years	3.15	0.62–15.92	0.164
Residence in Faridabad (ref: Delhi)	1.42	0.60–3.36	0.427
Extrapulmonary site (ref: pulmonary)	2.29	0.48–10.98	0.298
Ever smoking (ref: never)	0.91	0.29–2.82	0.866
Never use of smokeless tobacco (ref: ever)	4	1.22–13.16	0.022
AFB microscopy performed	2.26	0.94–5.43	0.068
CBNAAT performed	0.23	0.06–0.86	0.029
Line probe assay performed	6.64	1.68–26.22	0.007
Cough	0.62	0.19–2.06	0.435
Haemoptysis	0.64	0.20–2.06	0.455
Chest pain	0.52	0.20–1.34	0.175
Fever	0.29	0.07–1.25	0.096
Weight loss	3.11	0.99–9.75	0.051
Enlarged lymph nodes	1.11	0.21–5.89	0.901

**Table 11:** Alternative definition — test variables anchored on sample-collection date N = 158; events = 95; non-events = 63; parameters = 16; events per variable = 3.9. CB-NAAT: Cartridge-based nucleic acid amplification test

Variable	aOR	95% CI	p-value
Age ≤18 years (ref: >50 years)	2.12	0.38–11.79	0.392
Age 19–30 years	2.06	0.42–10.05	0.373
Age 31–50 years	2.76	0.55–13.85	0.217
Residence in Faridabad (ref: Delhi)	1.59	0.67–3.74	0.29
Extrapulmonary site (ref: pulmonary)	2.37	0.49–11.33	0.281
Ever smoking (ref: never)	0.8	0.25–2.56	0.708
Never use of smokeless tobacco (ref: ever)	4.09	1.24–13.51	0.021
AFB microscopy performed	2.49	1.06–5.87	0.037
CBNAAT performed	0.32	0.09–1.10	0.071
Line probe assay performed	5.03	1.77–14.25	0.002
Cough	0.49	0.15–1.66	0.254
Haemoptysis	0.79	0.26–2.45	0.689
Chest pain	0.52	0.21–1.31	0.167
Fever	0.24	0.05–1.09	0.065
Weight loss	3.95	1.23–12.74	0.021
Enlarged lymph nodes	1.03	0.20–5.30	0.968

Counts under the two definitions. Result on or before diagnosis (Table 10): AFB microscopy 63, CBNAAT 130, line probe assay 35, culture 2. Sample collected on or before diagnosis (Table 11): AFB microscopy 64, CBNAAT 132, line probe assay 46, culture 5. The collection-date definition counts assays whose results arrived after the diagnosis and so could not have informed it, which is why it is not used in the main analysis. The estimates are similar under both.

Firth Penalised-Likelihood Re-estimation of the Full Diagnostic-Delay Model

**Table 12:** Diagnostic delay — Firth penalised logistic regression (submitted exposure window) N = 158; penalised log likelihood = −68.46; Wald chi2(16) = 28.49, p = 0.028.

Variable	aOR (Firth)	95% CI	p-value
Age ≤18 years (ref: >50 years)	2.34	0.47–11.52	0.297
Age 19–30 years	2.15	0.50–9.36	0.306
Age 31–50 years	2.68	0.61–11.85	0.194
Residence in Faridabad (ref: Delhi)	1.36	0.60–3.05	0.461
Extrapulmonary site (ref: pulmonary)	2.05	0.49–8.54	0.322
Ever smoking (ref: never)	0.92	0.32–2.65	0.884
Never use of smokeless tobacco (ref: ever)	3.32	1.11–9.92	0.032
AFB microscopy performed	2.06	0.90–4.68	0.086
CBNAAT performed	0.29	0.09–0.95	0.041
Line probe assay performed	4.91	1.44–16.80	0.011
Cough	0.66	0.22–2.02	0.469
Haemoptysis	0.67	0.23–2.01	0.48
Chest pain	0.57	0.24–1.36	0.204
Fever	0.35	0.09–1.36	0.13
Weight loss	2.65	0.92–7.59	0.07
Enlarged lymph nodes	1.1	0.24–5.07	0.903

**Table 13:** Treatment initiation delay — Firth penalised logistic regression N = 158; penalised log likelihood = −82.64; Wald chi2(9) = 15.50, p = 0.078.

Variable	aOR (Firth)	95% CI	p-value
Age ≤18 years (ref: >50 years)	5.2	0.96–28.04	0.055
Age 19–30 years	3.31	0.67–16.46	0.143
Age 31–50 years	3.58	0.71–18.14	0.123
Residence in Faridabad (ref: Delhi)	2.84	1.38–5.84	0.005
Education 6–12 years (ref: ≤5 years)	0.85	0.37–1.99	0.714
Education >12 years	0.43	0.13–1.40	0.162
Formal employees and sales workers (ref: unemployed)	0.71	0.23–2.12	0.534
Manual workers and elementary occupations	0.58	0.22–1.49	0.255
Extrapulmonary site (ref: pulmonary)	2.39	0.95–6.01	0.063

Estimated with firthlogit in Stata 17.0. Under penalisation the paediatric association no longer reaches conventional significance. Exact logistic regression restricted to the ≤18 and >50 year groups agrees: odds ratio 4.83 (95% CI 0.90-49.76, p = 0.072).
